# Serum Concentrations of Selected Persistent Organic Pollutants in a Sample of Pregnant Females and Changes in Their Concentrations during Gestation

**DOI:** 10.1289/ehp.0800105

**Published:** 2009-03-12

**Authors:** Richard Y. Wang, Ram B. Jain, Amy F. Wolkin, Carol H. Rubin, Larry L. Needham

**Affiliations:** 1 National Center for Environmental Health and; 2 National Center for Zoonotic, Vector-Borne, and Enteric Diseases, Centers for Disease Control and Prevention, Atlanta, Georgia, USA

**Keywords:** dioxin, females, organochlorine pesticides, PCB, persistent organic pollutants, pregnant, serum concentrations

## Abstract

**Objectives:**

In this study we evaluated the concentrations of selected persistent organic pollutants in a sample of first-time pregnant females residing in the United States and assessed differences in these concentrations in all pregnant females during gestation.

**Methods:**

We reviewed demographic and laboratory data for pregnant females participating in the National Health and Nutrition Examination Survey, including concentrations of 25 polychlorinated biphenyls (PCBs), 6 polychlorinated dibenzo-*p*-dioxins (PCDDs), 9 polychlorinated dibenzofurans (PCDFs), and 9 organochlorine pesticides. We report serum concentrations for first-time pregnant females (2001–2002; *n* = 49) and evaluate these concentrations in all pregnant females by trimester (1999–2002; *n* = 203) using a cross-sectional analysis.

**Results:**

The chemicals with ≥ 60% detection included PCBs (congeners 126, 138/158, 153, 180), PCDDs/PCDFs [1,2,3,4,6,7,8-heptachlorodibenzo-*p*-dioxin (1234678HpCDD), 1,2,3,6,7,8-hexachlorodibenzo-*p*-dioxin (123678HxCDD), 1,2,3,4,6,7,8-heptachlorodibenzofuran (1234678HpCDF), 1,1′-(2,2-dichloroethenylidene)-bis(4-chlorobenzene) (*p*,*p*′-DDE)], and *trans*-nonachlor. The geometric mean concentration (95% confidence intervals) for 1234678HpCDD was 15.9 pg/g lipid (5.0–50.6 pg/g); for 123678HxCDD, 9.7 pg/g (5.5–17.1 pg/g); and for 1234678HpCDF, 5.4 pg/g (3.3–8.7 pg/g). The differences in concentrations of these chemicals by trimester were better accounted for with the use of lipid-adjusted units than with whole-weight units; however, the increase in the third-trimester concentration was greater for PCDDs/PCDFs (123678HxCDD, 1234678HpCDF) than for the highest concentration of indicator PCBs (138/158, 153, 180), even after adjusting for potential confounders.

**Conclusion:**

The concentrations of these persistent organic pollutants in a sample of first-time pregnant females living in the United States suggest a decline in exposures to these chemicals since their ban or restricted use and emission. The redistribution of body burden for these and other persistent organic pollutants during pregnancy needs to be more carefully defined to improve the assessment of fetal exposure to them based on maternal serum concentrations. Additional studies are needed to further the understanding of the potential health consequences to the fetus from persistent organic pollutants.

Persistent organic pollutants are chemicals with notable environmental persistence and the potential for long-range transport, bio-accumulation, and toxic effects. In 2001, the Stockholm Convention identified 12 of these chemicals, including polychlorinated biphenyls (PCBs), polychlorinated dibenzo-*p*-dioxins (PCDDs), polychlorinated dibenzofurans (PCDFs), and selected organochlorine pesticides such as dichlorodiphenyltrichloroethane (DDT) (United Nations Environment Program 2001). Although the use and emission of these chemicals in the United States were discontinued or restricted in the mid- to late 1970s, the general population is still being exposed to them at low levels, predominantly by the dietary route [Centers for Disease Control and Prevention (CDC) 2005a].

The pregnant woman’s exposure to persistent organic chemicals is of much interest because of exposures to the fetus and breast-fed infant and potential subsequent health effects. During pregnancy, the disposition of chemicals can be affected by physiologic changes, such as increased renal perfusion, increased volume of distribution, and increased serum lipids. The 200–400% increase of serum triglyceride concentration (Montes et al. 1984; Sattar et al. 1997) can cause the redistribution of lipophilic chemicals stored in adipose tissue to the blood compartment (Phillips et al. 1989). The significance of understanding the changes of these chemicals during gestation is to appropriately classify the fetus’s level of exposure based on the sampling period during gestation (Longnecker et al. 1999), and to determine whether there are different risks to the fetus based on the exposure to these chemicals during certain gestational periods. Previous studies with pregnant females have demonstrated inconsistent trends of blood concentrations of PCBs and selected organochlorine pesticides [1,1′-(2,2-dichloroethenylidene)-bis(4-chlorobenzene) (*p*,*p*′-DDE)], *trans*-nonachlor, hexachlorobenzene, and β-hexacyclochlorohexane (β-HCH)] throughout the trimesters (Jarrell et al. 2005; Longnecker et al. 1999; Takser et al. 2005). Potential delays in growth and neurodevelopment in the fetus and breast-fed infant from exposure to persistent organic pollutants (Jacobson and Jacobson 1996; Koopman-Esseboom et al. 1994; Patandin et al. 1999; Siddiqui et al. 2003) have been studied; the results suggest that these health effects can be related to late gestational exposure, although these findings are inconclusive, and additional studies are necessary to better characterize these risks to the newborn. Because these chemicals tend to accumulate in the body over a person’s lifetime, women of childbearing age should consider their exposure to these chemicals as they plan their reproductive future.

First-time pregnant females are considered a reliable index of exposure among pregnant females because of the lack of potential confounding variables, such as parity and prior breast-feeding (James et al. 2002; Sarcinelli et al. 2003), and they have been used in international studies to monitor the entire population’s exposure to these chemicals [World Health Organization (WHO) 2007]. We evaluated the concentrations of persistent organic pollutants in a sample of first-time pregnant females residing in the United States and the changes of these concentrations during gestation for all pregnant women participating in National Health and Nutrition Examination Survey (NHANES) 1999–2002.

## Methods

We selected participants in this study from NHANES (1999–2000–2001–2002), which used a complex, stratified, multistage, probability sampling designed to be representative of the civilian, noninstitutionalized U.S. population based on age, sex, and race/ethnicity (CDC 2004). We selected all females 16–59 years of age from these two survey periods and categorized them as pregnant or nonpregnant based on a urine pregnancy test that was used to screen participants for exclusion from a dual-energy X-ray absorptiometry scan. We analyzed the data for clinical chemistries and selected PCDDs, PCDFs, non-*ortho*-substituted or coplanar PCBs, other PCBs, organochlorine pesticides, and selected insecticide metabolites. The environmental chemicals were measured in serum by gas chromatography/isotope-dilution high-resolution mass spectrometry (CDC 2005b,2005c). We calculated serum concentration of total lipids using the concentrations for triglycerides and total cholesterol measured by an enzymatic method: total lipids = (2.27 × total cholesterol) + triglycerides + 62.3 mg/dL (Phillips et al. 1989).

We performed all analyses using SAS (version 9.1; SAS Institute Inc., Cary, NC) and SUDAAN (RTI International, Research Triangle Park, NC). We adjusted geometric means (GMs), percentages, and their confidence intervals for complex survey design as suggested by Korn and Grubbard (1998). We determined the GM for a chemical when the frequency of detection was at least 60%. We did not report the 50th percentile estimate (median) when it was less than the maximum limit of detection (LOD) for this study. We fitted regression models for each chemical for all females and pregnant females for all 4 years of data, but for first-time pregnant females we used data from only the 2001–2002 survey because of the difference in the LOD for these chemicals between the two survey periods. We used the LOD divided by the square root of 2 to impute a value for a chemical with a concentration less than LOD. We used SUDAAN Proc REGRESS in general linear models to calculate the adjusted GM. We used log-transformed values of each chemical as a dependent variable. Covariates and independent variables used were based on existing knowledge about these chemicals and the study population. These variables included age as a continuous variable, race/ethnicity (non-Hispanic whites, non-Hispanic blacks, Mexican Americans, and remaining groups), country of birth (United States, non-United States), number of children breast-fed for at least 1 month, fish and seafood consumed in the last 30 days, serum cotinine, body mass index (BMI), and parity, categorized as nulliparous (having borne no viable fetuses and not pregnant), primiparous (bearing or having borne one viable fetus), and multiparous (having had two or more pregnancies resulting in viable fetuses). We used trimester as a covariate in models for pregnant and first-time pregnant females and defined it as follows: first trimester, < 4 months; second trimester, 4–6 months; and third trimester, > 6 months. By analyzing the cross-sectional data for these females at different trimesters, these results would approximate those observed by following these females longitudinally.

## Results

### Study population of all participants

Table 1 presents data for the female participants in this study by their age, race/ethnicity, parity, pregnancy status, and stage of gestation by trimester. The median ages in years for all females in this study by parity were, for nulliparous, 18.0; primiparous, 26.0; and multiparous, 41.0 (data not shown). The minimum and maximum ages for first-time pregnant females and all pregnant females in this study were 16–38 and 16–50 years, respectively. Only one participant was 50 years of age.

The pregnant females demonstrated differences in physiologic parameters that were consistent with their gestational stage by trimester, which supported the use of these females as a model for gestation. For example, we observed the GMs for the following variables by trimester (first, second, third): BMI (27.03 kg/m^2^, 28.52 kg/m^2^, 31.1 kg/m^2^), serum triglycerides (102.43 mg/dL, 148.19 mg/dL, 185.38 mg/dL), serum total lipids (593.91 mg/dL, 701.60 mg/dL, 790.75 mg/dL), hemoglobin (12.88 g/dL, 11.96 g/dL, 11.91 g/dL), serum albumin (3.98 g/dL, 3.67 g/dL, 3.30 g/dL), and serum creatinine (0.53 mg/dL, 0.43 mg/dL, 0.45 mg/dL). The apparent progressive increase in BMI throughout gestation is primarily attributed to increases in total body water and adipose tissue. Increased total body water accounted for the dilutional effect seen with hemoglobin and serum albumin concentrations, and increased cardiac output and renal perfusion led to increased creatinine clearance. During the second and third trimesters, fetal growth and the preparation for lactation caused increased lipid metabolism and whole-weight serum concentrations in these females. The serum concentrations (whole weight) of several of the chemicals measured in this study directly correlated with those of serum lipids; the triglycerides were better correlated with PCDDs/PCDFs than with PCBs [see Supplemental Material, Table 1 (doi: 10.1289/ehp.0800319.S1)].

When we compared whole-weight and lipid-adjusted concentrations of these chemicals by trimesters in all pregnant females, the amount of change in the concentrations among trimesters was greater for whole-weight units than for lipid-adjusted units [see Supplemental Material, Table 2 (doi: 10.1289/ehp.0800319.S1)]. For example, the range for the absolute difference in the GM concentrations of the indicator PCBs (138/158, 153, 180) between the trimesters were for first and third trimesters, whole weight, 20–33%, versus lipid adjusted, 3–7%; and for second and third trimesters, whole weight, 43–60%, versus lipid adjusted, 24–35%. For PCDDs/PCDFs, these differences were for first and third trimesters, whole weight, 23–90%, versus lipid adjusted, 8–51%; and for second and third trimesters, whole weight, 43–87%, versus lipid adjusted, 31–74%. These comparisons demonstrated that lipid adjustment accounted for the change in the chemical concentrations during gestation better than did the use of whole-weight units. Thus, we used lipid adjusted concentrations in the mathematical models.

Among the variables in the general linear model for all pregnant females, the concentrations of several of the chemicals increased with age, except for 1,2,3,4,6,7,8-heptachlorodibenzofuran (1234678HpCDF) and β-HCH [see Supplemental Material, Table 3 (doi: 10.1289/ehp.0800319.S1)]. For BMI, only the PCBs were inversely related to BMI (Table 2), which also has been previously observed in pregnant females (Glynn et al. 2007; James et al. 2002; Wolff et al. 2005). Among the various race/ethnicities, the concentrations of several chemicals, including indicator PCBs, PCDDs/PCDFs, and organochlorine pesticides, were higher in non-Hispanic blacks than in non-Hispanic whites in all females in this study (Table 3). However, Mexican Americans had lower indicator PCB concentrations (e.g., 20% lower for PCB-153 based on adjusted GM) than did non-Hispanic whites. Females of non-U.S. birth had lower concentrations of 1,2,3,6,7,8-hexachlorodibenzo-*p*-dioxin (123678HxCDD), octachlorodibenzo-*p*-dioxin (OCDD), and 1234678HpCDF and higher concentrations of indicator PCBs (congeners 153, 180) and β-HCH than did females of U.S. birth (Table 3). The dietary intake of fish or shellfish in the last 30 days was associated with increased concentrations of PCB-153, PCB-169, and 1234678HpCDF [see Supplemental Material, Table 6 (doi: 10.1289/ehp.0800319.S1)].

### First-time pregnant females

Table 4 lists the chemicals that were detected in at least 10% of the first-time pregnant females sampled in 2001–2002. We determined the GMs or medians of the serum concentrations of four PCBs, three PCDDs/PCDFs, and two organochlorine pesticides, metabolites, or degradates; these chemicals consisted of hexa- and heptachlorinated PCBs and PCDDs/PCDFs, *p*,*p*′-DDE, and *trans*-nonachlor. For DDT, the parent chemical *p*,*p*′-DDT and its environmental degradate, *p*,*p*′-DDE, were detected in 12.8% and 100% of these females, respectively. The commonly detected (> 50%) PCDDs/PCDFs were 123678HxCDD (80%), 1,2,3,4,6,7,8-heptachlorodibenzo-*p*-dioxin (1234678HpCDD; 100%), 1234678HpCDF (90%), and OCDD (51.5%).

The PCBs detected in > 10% of this group of females ranged in order of chlorination from tetrachlorinated to heptachlorinated biphenyl homologs, and this distribution is consistent with the major congeners found in Aroclor mixtures (e.g., 1254 and 1260) that were widely used in the United States in the mid-1970s. The ranges of the frequency of detection and GMs of the indicator PCBs (congeners 138/158, 153, 180) were 77–89% and 6.4–9.8 ng/g lipid adjusted, respectively (Table 4). The intercorrelations between these indicator PCBs based on lipid-adjusted concentrations in these females were as follows: PCBs 138/158:153, *r* = 0.99, *p* < 0.0001; PCBs 153:180, *r* = 0.99, *p* < 0.0001; and PCBs 180:138, *r* = 0.97, *p* < 0.0001 (data not shown). PCB-153 has been used as a general indicator for the exposure to non-dioxin-like PCBs and a reported correlation of 0.90 to total PCBs (Grimvall et al. 1997). The remaining non-dioxin-like PCBs measured in this study had a frequency of detection that ranged from 11.3% for PCB-187 to 34.9% for PCB-52 (Table 4). The detection frequencies for coplanar PCBs (congeners 126 and 169) and mono-*ortho* chlorinated PCBs (congeners 74 and 118) were about 50%, except for PCB-126, which was 93%.

### Trimesters and parity

The general pattern of the concentrations of several of these chemicals by trimesters was that they essentially remained unchanged during the first two trimesters and then increased from the second to the third trimester in the adjusted model (Table 2). The increase in the concentration of these chemicals during the last trimester was statistically significant for the PCDDs/PCDFs, but not for the indicator PCBs. When we adjusted for BMI and other potential confounding variables in the comparison of these concentrations among trimesters, the differences between the last two trimesters based on the adjusted GMs for 123678HxCDD and 1234678HpCDF were 52% and 30% (*p* < 0.05), respectively (Table 2). However, we observed essentially no differences for the indicator PCB concentrations (1–11%, *p*> 0.05) among these trimesters.

We evaluated parity in all pregnant females and the concentrations of several chemicals, including indicator PCBs (congeners 138/158, 153, 180), OCDD, and 1234678HpCDF, were lower in multiparous females than in primiparous females (Table 2). In the model including all females, nulliparous females had higher concentrations of *p*,*p*′-DDE and PCB-180 than did primiparous or multiparous females, and an increase in the number of children breast-fed for at least 1 month was associated with a lower concentration of indicator PCBs (Table 5).

## Discussion

This convenience sample of first-time pregnant females living in the United Sates in 2001–2002 includes the initial report of PCDDs/PCDFs in these females.

### Exposure levels

Overall, the concentrations of these chemicals in this study suggest a decline in the exposure to persistent organic pollutants in pregnant females residing in the United States since their ban or restricted use and emission in the late 1970s, which is consistent with the observed trend for the general population in previous reports (Patterson et al. 1994; CDC 2005a). More specifically, the concentrations of the indicator PCBs (congeners 138/150, 153, and 180) in first-time pregnant females in this study are lower than those reported for pregnant females who participated in the Collaborative Perinatal Project from 1959 through 1966 (Niswander and Gordoni 1972). In an approximately a one-third subset of first-time pregnant females, their median serum concentrations of PCB-153 was 140 ng/g lipid adjusted (Daniels et al. 2003) (vs. 8.0 ng/g lipid adjusted for this study) and for *p*,*p*′-DDE was 25 ng/mL whole weight (Longnecker et al. 2001) (vs. 113.0 ng/g lipid adjusted for this study). The rate of decline in the PCB-153 concentration from 1959–1966 to 1999–2002 appears to be consistent with the estimated half-life of approximately 10 years for these chemicals (Yakushij et al. 1984). The concentrations of PCDDs/PCDFs in adipose tissue were reported for females in the National Human Adipose Tissue Survey from 1986 to mid-1990 (Orban et al. 1994; Reynolds et al. 2005) and comparative data on these chemicals suggest a more gradual rate of decline than for the PCBs.

Human milk concentrations of these chemicals were measured in two pooled collections obtained from first-time mothers in the United States in 2002–2003 as part of the third round of the WHO-coordinated exposure study on the levels of PCBs, PCDDs, and PCDFs in human milk (PCB-153, 15.9 and 24.2 ng/g lipid; 1234678HpCDD, 16.0 and 12.9 pg/g lipid; 1234678HpCDF, 3.1 and 1.5 pg/g lipid; *p*,*p*′-DDE, 248 and 123 ng/g lipid) (Wang and Needham 2003, 2004). These concentrations are comparable to those for the first-time pregnant females in this study and lower than those measured in two pooled milk samples from U.S. women participating in a similar WHO survey conducted in 1989 (PCB-153, 80 and 88 ng/g lipid; 1234678HpCDD, 34 and 50 pg/g lipid; 1234678HpCDF, 2.4 and 5.7 pg/g lipid) (Yrjanheikki 1989). The direct comparison of concentrations of higher order chlorinated PCDDs/PCDFs of serum to milk specimens is limited by the diminished ability of these chemicals to transfer from blood to milk due to size exclusion (Wittsiepe et al. 2007). Thus, these findings support the use of first-time pregnant females and human milk as a matrix to monitor the population’s trend in exposure to persistent lipophilic chemicals, although the exposure level only reflects that of females of reproductive age (Wang et al. 2005). The added benefit of using milk as an exposure matrix includes the ability to assess the breast-fed infant’s intake of these chemicals.

Maternal parity, age, BMI, prior breast-feeding, and dietary intake of fish and shellfish are predictors for the serum concentrations of these chemicals, and differences in exposure levels exist among race/ethnicities. Differences in the concentrations of PCBs among groups in this study are likely attributable to variations in the dietary intake of fatty foods and the exposure to different PCB mixtures, such as the Aroclor mixtures (1260, 1254, 1248), which were produced and used in the United States. Environmental degradation and biologic metabolism of higher to lower chlorinated homologs can account for differences in PCB concentrations (e.g., PCB-169) in the population as well. In North America, the historic difference in the production and use of PCBs and DDT between Mexico and the United States may account for variations in the concentrations of these chemical in these populations. For example, Mexico imported but did not produce PCBs and was until recently using DDT to control vectors for malaria (Chanon et al. 2003). These practices are unlike those for the United States and may account for the low indicator PCB concentrations and high *p*,*p*′-DDE concentration in the Mexican Americans in this study. Other studies have demonstrated a difference in the concentrations of PCBs and *p*,*p*′-DDE in pregnant females (Bradman et al. 2007; James et al. 2002; Wolff et al. 2005) and in other groups in the population (Finklea et al. 1972; Rogan et al. 1986) based on race/ethnicity in the United States. The higher *p*,*p*′-DDE concentration in Mexican Americans than other race/ethnic groups in this study is similar to the findings in NHANES (2001–2002) (CDC 2005a) and Hispanic Health and Nutrition Examination Survey (1982–1984) (Akkina et al. 2004).

The increased concentrations of PCBs (congeners 153 and 169) and 1234678HpCDF in females who consumed a fish or a shellfish meal in the last 30 days of the survey in this study are consistent with prior reports demonstrating that these food groups can be a source of exposure to these chemicals in pregnant females and other groups in the population from regions known for their increased dietary intake of seafood, such as the Nordic countries and their territories (Glynn et al. 2007; Johansen et al. 1996) and the U.S. Great Lakes (Turyk et al. 2006). The increased consumption of crustaceans from contaminated waterways has been associated with a higher toxic equivalency concentration for PCDFs than for PCDDs, and the sum of these concentrations was higher than that for dioxin-like PCBs (Johansen et al. 1996).

### Trimesters

The serum whole-weight concentrations of these chemicals are higher in the third trimester than in earlier trimesters, and this is attributed to the progressive increase in maternal serum nonpolar lipids, such as triglycerides and cholesterol esters, that occurs throughout the latter two-thirds of gestation and is primarily for milk production (Sattar et al. 1997). The apparent differences in serum concentrations of these chemicals among the trimesters in this study are consistent with the physiologic changes during gestation, which include the body’s increasing volume of distribution and serum lipid concentrations. Increased body weight and BMI have been associated with decreased concentrations of PCBs and selected organochlorine pesticides during gestation (Glynn et al. 2007; James et al. 2002; Wolff et al. 2005). For example, Glynn et al. (2007) reported a decrease in the concentrations of PCBs and selected organochlorine pesticides of 16–38% per unit of body weight during gestation. These changes are likely explanations for the apparent decrease in the lipid-adjusted concentrations for PCBs (congeners 169 and 180) and PCDDs (1234678HpCDD) during the second trimester among all pregnant females in this study [see Supplemental Material, Table 2 (doi: 10.1289/ehp.0800319.S1)].

The nearly complete correction of the difference in the concentrations of the indicator PCBs between the first and the third trimester in this study by using lipid-adjusted units suggests that the increase in the serum PCB concentration reflects the redistribution of PCB from tissue stores (Gallenberg et al. 1987; Phillips et al. 1989). This observation has been demonstrated for serum total PCB concentrations in a longitudinal study of 67 pregnant females throughout their pregnancy (Longnecker et al. 1999). These changes in PCB concentrations among trimesters are presumed small because other studies of pregnant females have demonstrated a lack of statistically significant difference between these concentrations (Jarrell et al. 2005; Takser et al. 2005).

Compared with the indicator PCBs (congeners 138/158, 153, and 180), the increases in PCDD/PCDF (123678HxCDD, 1234678HpCDF) concentrations during the third trimester were greater, even after adjusting for serum total lipids and potential confounders in this study. These findings, in conjunction with the variation in correlations to serum lipids for these chemicals, suggest there is a difference in the interaction between these classes of chemicals and lipoproteins based on their lipid content that can be related to the gestational change in the serum lipid profile (Sattar et al. 1997). The increase in serum concentrations of nonpolar lipids can cause a greater redistribution of PCDDs/PCDFs than PCBs based on the difference in their dissolution in lipids over the duration of the gestation. The preferential affinity to triglyceride-rich lipoproteins, such as very low-density lipoprotein (VLDL), by PCDDs/PCDFs (Arehart et al. 2004; Marinovich et al. 1983) and PCBs (Gallenberg et al. 1987) has been demonstrated in various settings. In an *in vitro* model, tetrachlorodibenzo-*p*-dioxin (TCDD) had a greater affinity to VLDL and low-density lipoprotein (LDL) than did PCBs (congeners 105 and 126) (Arehart et al. 2004), which would be consistent with the difference in correlations with triglyceride between PCDDs/PCDFs and PCBs [see Supplemental Material, Table 1 (doi: 10.1289/ehp.0800319. S1)]. Further investigations are needed to better characterize the contributions of lipid solubility (Sandermann 2003), protein binding (e.g., apolipoprotein) (Arehart et al. 2004; Patterson et al. 1989), and molecular rigidity to the differences in gestational trend between PCDDs/PCDFs and PCBs observed in this study.

### Parity

The apparent decrease in concentrations of the persistent organic pollutants with increased parity may be attributed to the depuration of these chemicals from successive breast-feedings (López-Carrillo et al. 2001; Sarcinelli et al. 2003) because the amount of these chemicals eliminated by the transplacental route is presumed to be less than the amount loss by lactation (Jacobson and Jacobson 1996), which is consistent with the associations between prior breast-feeding and lower indicator PCB concentrations in this study (Table 5). However, the contribution to lower concentrations of these chemicals in females from their deposition in placental tissue or fetal tissues and organs cannot be excluded because parity remained a predictive variable for certain chemicals after adjusting for breast-feeding. The small changes in serum concentrations for certain PCDDs/PCDFs (e.g., 1234678HpCDF, 12346789OCDD) associated with prior breast-feeding among all females in this study might be attributable to the poor diffusion of these chemicals across the milk–blood barrier (Wittsiepe et al. 2007). These effects of breast-feeding and parity on the chemical concentrations support the use of first-time pregnant females as a reliable index of exposure to these chemicals among pregnant females.

### Limitations

The findings in this study are limited by the cross-sectional design, use of self-reported data for reproductive history, and small sample size. NHANES is designed to achieve representative concentrations of these environmental chemicals in the general U.S. population by age, sex, and race/ethnicity. Thus, the estimates of the central tendencies for these chemicals in first-time pregnant females in this study are likely to be unstable and not representative of this group in the general population because the statistical weighting factor used in this analysis was not designed for these females. Also, the observed gestational trends for these chemical concentrations need to be verified in a prospective observational study because their prepregnancy values might have biased the findings despite the use of statistical adjustments for potential confounders. Finally, the statistical power of the analyses to identify significant findings might have been limited because of the sample sizes, which could not be controlled because of the nature of the study design.

## Conclusions

The findings from this study suggest a decline in the exposure to these persistent organic pollutants in pregnant females in the United States since their ban or restricted use and emission, which is consistent with the exposure trend for these chemicals in the general population based on past reports. The redistribution of the body burden for persistent organic pollutants in response to the change in serum lipid profile during gestation needs to be more carefully defined because the concentrations of some of these chemicals can vary on a whole-weight or lipid-adjusted basis during this period. The sampling of blood from pregnant females at a specified time during gestation and the use of lipid-adjusted units can minimize these differences and improve the interpretation of the fetus’s level of exposure to these chemicals based on maternal serum concentrations. Additional studies on the exposure and disposition of these and other persistent organic pollutants in pregnant females are needed to better understand the potential health consequences to the fetus from these exposures.

## Figures and Tables

**Table 1:** Unweighted sample sizes (no.) for pregnant and nonpregnant females in this study by age, race/ethnicity, parity, and gestational trimester.

Characteristic	All females	All pregnant females	First-time pregnant females
Total	1,584	203	49
Age (years)			
16–19	534	21	7
20–29	326	122	31
30–59	724	60	11
Race/ethnicity			
Non-Hispanic white	611	92	26
Non-Hispanic black	352	30	5
Mexican American	462	54	13
Remaining groups	159	27	5
Parity			
Nulliparous	705	—	—
Primiparous	337	155	49
Multiparous	542	48	—
Trimester			
First		78	26
Second		57	15
Third		68	8

Data are for all females (16–59 years of age) who had a urine pregnancy test in NHANES 1999–2000 and 2001–2002, all pregnant females based on a urine pregnancy test in NHANES 1999–2000 and 2001–2002, and first-time pregnant females based on a urine pregnancy test in NHANES 2001–2002.

**Table 2:** Adjusted GM (95% confidence interval) for concentrations (lipid adjusted) of persistent organic pollutants by parity and trimester and regression coefficients for BMI for all pregnant females in this study, identified from NHANES 1999–2000 and 2001–2002.

Chemical	Parity		Trimester			BMI β (SE)
	Primiparous	Multiparous	First	Second	Third	
PCB-126 (pg/g)	11.7 (9.1–14.9)	7.5 (3.9–14.5)	9.3 (6.4–13.6)	10.5 (7.4–15.0)	12.0 (7.8–18.7)	−0.001 (0.007)
PCB-138/158 (ng/g)	11.2 (9.8–12.8)	6.1 (4.5–8.3)**	9.7 (8.5–11.1)	9.0 (7.7–10.6)	9.8 (8.0–12.0)	−0.008 (0.004)*
PCB-153 (ng/g)	14.7 (12.4–17.5)	7.6 (5.3–10.7)**	12.1 (10.5–13.9)	11.8 (10.0–13.9)	13.1 (10.4–16.5)	−0.011 (0.004)*
PCB-169 (pg/g)	6.6 (5.6–7.8)	4.2 (2.7–6.6)	5.8 (4.5–7.3)	4.9 (3.8–6.3)	7.0 (5.8–8.5)#	−0.013 (0.005)*
PCB-180 (ng/g)	8.3 (7.1–9.8)	4.5 (3.3–6.0)**	7.1 (6.0–8.4)	6.9 (5.9–8.0)	7.0 (5.6–8.7)	−0.01 (0.004)*
123678HxCDD (pg/g)	10.8 (8.7–13.4)	7.6 (5.1–11.3)	8.9 (7.2–11.0)	8.1 (6.3–10.4)	13.4 (9.9–18.3)#	0.008 (0.005)
1234678HpCDD (pg/g)	31.7 (24.4–41.2)	21.4 (15.7–29.1)	31.9 (24.7–41.1)	23.4 (17.9–30.5)	28.5 (19.2–42.2)	0.008 (0.005)
12346789OCDD (pg/g)	274.4 (219.7–342.7)	129.3 (93.5–178.6)**	217.5 (159.8–296.0)	208.3 (173.1–250.7)	252.7 (180.1–354.5)	0.004 (0.004)
1234678HpCDF (pg/g)	8.1 (6.8–9.7)	3.8 (2.7–5.4)**	6.2 (5.1–7.6)	5.9 (4.8–7.2)	8.1 (6.6–9.9)#	0.006 (0.005)
β-HCH (ng/g)	4.6 (3.7–5.9)	6.2 (2.1–18.5)	5.2 (3.8–7.2)	6.6 (3.6–12.0)	3.7 (2.9–4.7)	0.007 (0.006)
*p*,*p*′-DDE (ng/g)	141.6 (115.0–174.4)	135.9 (66.4–278.2)	117.6 (82.4–168.0)	163.1 (110.7–240.2)	159.1 (128.5–196.8)	−0.013 (0.005)
*trans*-Nonachlor (ng/g)	8.9 (7.7–10.4)	7.5 (4.9–11.4)	8.1 (6.8–9.8)	7.9 (6.4–9.7)	9.9 (7.7–12.8)	−0.013 (0.004)

Sample size varied from 147 to 179, depending on the chemical. The model was adjusted for parity, trimester, age, BMI, race/ethnicity, U.S. birth, fish and shellfish eaten in the last 30 days, number of children breast-fed ≥ 1 month, and serum cotinine as independent variables. Log of serum concentration (lipid adjusted) of the chemical was the dependent variable. Remaining data for the model are provided in Supplemental Material, Tables 3–5 (doi: 10.1289/ehp.0800319.S1).

* Statistically significantly different from 0 at α = 0.05.

** Statistically significantly different from primiparous females at α = 0.05.

# Statistically significantly different from second trimester at α = 0.05.

**Table 3:** Adjusted GM (95% confidence interval) for concentrations (lipid adjusted) of persistent organic pollutants by race/ethnicity and country of birth for all females in this study, identified from NHANES 1999–2000 and 2001–2002.

	Race/ethnicity			Country of birth	
Chemical	Non-Hispanic white	Non-Hispanic black	Mexican American	United States	Non-United States
PCB-126 (pg/g)	13.9 (12.4–15.6)	20.3 (16.9–24.5)*	15.9 (14.2–17.7)	19.5 (17.2–22.2)	13.6 (12–15.4)
PCB-138/158 (ng/g)	16.2 (15.1–17.3)	21.7 (19.4–24.2)*	13.9 (12.6–15.4)*	15.7 (14.1–17. 4)	17.4 (16.2–18.6)
PCB-153 (ng/g)	22.8 (21.5–24.2)	30.5 (28–33.2)*	18.2 (16.5–20)*	20.8 (19–22.9)	25.1 (23.5–26.7)**
PCB-169 (pg/g)	10.9 (9.9–12)	13.4 (12.1–14.9)*	9.4 (8.7–10.2)*	11.8 (10.7–12.9)	10.8 (9.8–11.9)
PCB-180 (ng/g)	14.1 (13.2–15)	17.2 (16.1–18.4)*	12.3 (11.5–13.2)*	13.9 (13.1–14.8)	14.5 (13.4–15.7)**
1234678HpCDD (pg/g)	30.9 (27.8–34.2)	44.2 (39.2–49.8)*	39.3 (35.3–43.9)*	34.5 (30–39.7)	31.9 (29–35.1)
123678HxCDD (pg/g)	14.1 (12.4–16)	20.4 (16.3–25.4)*	16.3 (14.7–18.2)	27.7 (24.7–31.1)	10.9 (9.3–12.8)**
1234678HpCDF (pg/g)	6.6 (5.9–7.3)	9.8 (8.7–11.1)*	6.7 (6.1–7.5)	9.5 (8.4–10.7)	5.8 (5.2–6.6)**
12346789OCDD (pg/g)	260 (235.3–287.3)	372 (333.2–415.2)*	324.2 (295.7–355.6)*	350.7 (310.7–395.7)	245.7 (221.4–272.7)**
β-HCH (ng/g)	6.7 (6.2–7.2)	7.3 (6.5–8.3)	19 (16–22.5)*	6.7 (6–7.5)	8.7 (8–9.6)**
*p*,*p*′-DDE (ng/g)	177.2 (156.7–200.3)	311.6 (253.2–383.4)*	806.8 (674.6–964.8)*	234.3 (204.8–268)	239.4 (213.1–268.9)
*trans*-Nonachlor (ng/g)	13.9 (12.7–15.2)	18.2 (16–20.8)*	14.8 (13.2–16.7)	13.9 (12.6–15.3)	15 (13.6–16.5)

Sample size varied from 779 to 1,088, depending on the chemical. The model was adjusted for parity, trimester, pregnancy status, age, BMI, race/ethnicity, country of birth, fish and shellfish eaten in the last 30 days, number of children breast-fed ≥ 1 month, and serum cotinine as independent variables. Log of serum concentration (lipid adjusted) chemical was the independent variable. Remaining data for the model are provided in Supplemental Material, Tables 6 and 7 (doi: 10.1289/ehp.0800319.S1).

* Statistically significantly different from non-Hispanic whites at α = 0.05.

** Statistically significantly different from those born in the United States at α = 0.05.

**Table 4:** Serum concentrations for persistent organic pollutants measured in first-time pregnant females in this study, identified from NHANES 2001–2002.

	Maximum LOD	Percent below LOD	GMs (95% CI)		Median (95% CI)Lipid adjusted
			Whole weight	Lipid adjusted	
Non-dioxin-like PCBs (ng/g)					
PCB-52	11.5	65.1	NC	NC	NC
PCB-99	7.6	80.1	NC	NC	NC
PCB-138/158	7.6	23.3	0.06 (0.04–0.07)	8.4 (6.2–11.3)	NC
PCB-153	7.6	19.8	0.06 (0.05–0.08)	9.8 (7.2–13.4)	8.0 (5.2–17.8)
PCB-170	7.6	71.8	NC	NC	NC
PCB-180	7.6	35	0.04 (0.03–0.06)	6.4 (4.5–9.2)	NC
PCB-187	7.6	88.7	NC	NC	NC

Coplanar PCBs (fg/g for whole weight, pg/g for lipid adjusted)					
PCB-126	9.2	7.3	101.5 (56.0–183.9)	15.7 (7.4–33.5)	11.2 (3.2–211.0)
PCB-169	7.2	53.1	NC	NC	NC

Mono-*ortho*-substituted PCBs (ng/g)					
PCB-74	7.6	50	NC	NC	NC
PCB-118	7.6	65.3	NC	NC	NC

PCDDs (fg/g for whole weight, pg/g for lipid adjusted)					
1234678HpCDD	—	0	104.4 (36.6–298.0)	15.9 (5.0–50.6)	12.4 (5.4–59.6)
123678HxCDD	6.4	19.7	64.0 (34.3–119.3)	9.7 (5.5–17.1)	NC
12346789OCDD	180	48.5	NC	NC	NC

PCDFs (fg/g for whole weight, pg/g for lipid adjusted)					
1234678HpCDF	3.7	10	35.2 (23.8–52.1)	5.4 (3.3–8.7)	4.3 (2.4–14.4)
123478HxCDF	4.2	58.1	NC	NC	NC
123678HxCDF	4.1	77.7	NC	NC	NC
23478PeCDF	5.5	71.2	NC	NC	NC

Organochlorine insecticides (ng/g)					
*p,p*′-DDE	—	0	1.2 (0.6–2.7)	186.2 (75.1–461.2)	113.0 (93.7–269.0)
*p,p*′-DDT	12.6	87.2	NC	NC	NC
Heptachlor epoxide	7.6	88.5	NC	NC	NC
β-HCH	7.6	73.8	NC	NC	NC
Oxychlordane	7.6	67.7	NC	NC	NC
*trans*-Nonachlor	5.9	23.1	0.05 (0.04–0.06)	7.4 (5.9–9.3)	7.4 (3.5–7.4)

Sample size varied from 23 to 39, depending on the chemical. Estimates were not calculated (NC) when either the proportion of results below the LOD was too high to provide a valid result or the estimate of the percentile was below the maximum LOD.

**Table 5:** Adjusted GM (95% confidence interval) for concentrations (lipid adjusted) of persistent organic pollutants and regression coefficient for number of breast-fed children for at least 1 month, parity, and pregnancy status for all females in this study, identified from NHANES 1999–2000 and 2001–2002.

Chemical	No. of children breast-fed ≥ 1 month β (SE)	Parity			Pregnant	
		Nulliparous	Primiparous	Multiparous	Yes	No
PCB-126	0.0045 (0.019)	16.7 (13.7–20.5)	16.6 (13.4–20.6)	14.6 (11.7–18.1)	12 (8.3–17.3)	15.9 (14.5–17.3)
PCB-138/158	−0.025 (0.012)##	17.4 (15.3–19.9)	17.1 (15.1–19.5)	15.8 (14–17.9)	13.3 (11.4–15.5)	16.8 (15.8–18)#
PCB-153	−0.026 (0.011)##	24.9 (22.4–27.6)	24.3 (21.6–27.3)	21.5 (19.4–23.9)	18.5 (15.8–21.6)	23.4 (22.1–24.9)#
PCB-169	−0.0155 (0.017)	11.5 (10–13.2)	11.8 (10.4–13.3)	10.7 (9.3–12.4)	8.3 (6.7–10.10)	11.4 (10.6–12.2)#
PCB-180	0.040 (0.011)##	15.8 (14.3–17.5)	14.9 (13.1–17)	12.9 (11.8–14.2)**	11.6 (10.2–13.3)	14.4 (13.6–15.2)#
1234678HpCDD	0.0036 (0.014)	32 (27.7–37)	33.7 (29.3–38.9)	33 (28.7–37.9)	29.1 (23.4–36.2)	33.1 (30.6–35.8)
123678HxCDD	−0.0287 (0.024)	11.5 (9.4–14.1)	15.3 (12.4–18.9)**	18.5 (15.3–22.2)**	11.7 (9.3–14.7)	15.8 (14.1–17.8)#
1234678HpCDF	0.0030 (0.012)	7.1 (6.2–8.2)	6.9 (5.9–8.1)	7.1 (6.1–8.2)	6.7 (5.4–8.3)	7.1 (6.5–7.7)
12346789OCDD	0.00017 (0.0.14)	272.2 (235–315.2)	263.7 (229.1–303.4)	291.8 (252.6–336.9)	278.5 (224.9–344.9)	280.2 (258.5–303.8)
β-HCH	−0.0155 (0.017)	8 (6.8–9.6)	7.3 (6.3–8.4)	7.8 (6.8–8.9)	7.3 (5–10.7)	7.8 (7.3–8.4)
*p*,*p*′DDE	−0.0152 (0.021)	280.6 (229.8–342.6)	214.3 (187.6–244.7)**	220.9 (192.5–253.6)	228.3 (172–303.1)	237.7 (217.6–259.7)
*trans*-Nonachlor	−0.0317 (0.016)	15 (13.2–17.1)	14.4 (12.8–16.1)	14.2 (12.6–16.1)	13.1 (11.3–15.2)	14.6 (13.6–15.6)

Sample size varied from 779 to 1,088, depending on the chemical. The model was adjusted for parity, trimester, pregnancy status, age, BMI, race/ethnicity, country of birth, fish and shellfish eaten in the last 30 days, number of children breast-fed ≥ 1 month, and serum cotinine as independent variables. Log of serum concentration (lipid adjusted) chemical was the independent variable. Remaining data for the model are provided in Supplemental Material, Tables 6 and 7 (doi: 10.1289/ehp.0800319.S1).

** Statistically significantly different than nulliparous females at α = 0.05.

# Statistically significantly different than pregnant females at α = 0.05.

## Statistically significantly different than 0 at α = 0.05.
